# Characterization of Oil Body and Starch Granule Dynamics in Developing Seeds of *Brassica napus*

**DOI:** 10.3390/ijms24044201

**Published:** 2023-02-20

**Authors:** Kang Chen, Yongtai Yin, Yiran Ding, Hongbo Chao, Maoteng Li

**Affiliations:** 1 Department of Biotechnology, College of Life Science and Technology, Huazhong University of Science and Technology, Wuhan 430074, China; 2 Key Laboratory of Molecular Biophysics of the Ministry of Education, Wuhan 430074, China; 3 School of Agricultural Sciences, Zhengzhou University, Zhengzhou 450001, China

**Keywords:** *Brassica napus*, oil body, starch granule, developing seeds

## Abstract

*Brassica napus* is the most important oilseed crop in the world, and the lipid was stored in the oil body (OB) in the form of triacylglycerol. At present, most of studies on the relationship between oil body morphology and seed oil content in *B. napus* was focused on mature seeds. In the present study, the OBs in different developing seeds of *B. napus* with relatively high oil content (HOC) of about 50% and low oil content (LOC) of about 39% were analyzed. It was revealed that the size of OBs was first increased and then decreased in both materials. And in late seed developmental stages, the average OB size of rapeseed with HOC was higher than that of LOC, while it was reversed in the early seed developmental stages. No significant difference was observed on starch granule (SG) size in HOC and LOC rapeseed. Further results indicated that the expression of genes that involved in malonyl-CoA metabolism, fatty acid carbon chain extension, lipid metabolism, and starch synthesis in the rapeseed with HOC was higher than that of rapeseed with LOC. These results give some new insight for understanding the dynamics of OBs and SGs in embryos of *B. napus.*

## 1. Introduction

Rapeseed (*Brassica napus*, AACC, 2n = 38) is the second-largest oilseed crop in the world, providing 13–16% of vegetable oil production [1,2,3]. With the increase in demand for vegetable oils used to produce food, energy, and bio-related products, the global demand gap for vegetable oils is also constantly growing [2,4]. Therefore, it is important to reveal the mechanism of oil accumulation, which could provide some new ideas for improving the oil content of rapeseed in the future.

Lipids are one of the most important storage substances in seeds and are usually stored in the form of triacylglycerol (TAG), which is the main component of the oil body (OB) [5,6,7,8,9]. OB is formed by semi-unit membranes wrapped around the liquid matrix TAG and is involved in many other cellular processes, such as stress responses, lipid metabolism, organ development and hormone signaling for this special construction [10,11,12,13,14]. The semi-unit membrane consists of a phospholipid monolayer and embedded structure protein, which is essential for lipid accumulation and OB stability. The structural proteins embedded in the semi-unit membrane mainly include oleosin, caleosin, and steroleosin, which play a crucial role in lipid accumulation, OB formation and OB movement [7,15,16,17,18,19]. Previous studies have revealed that the diameter of mature OBs ranges from 0.5 to 2.5 µm, and the relationship between OB size, OB morphology and oil content (OC) has also been studied in different species [6,20,21]. In maize, varieties with HOC possess relatively larger OBs compared to maize varieties with LOC [16]. In tung trees, the size of OBs in endosperm gradually decreases with the increase of OC, but the number of OBs increases [22]. In rapeseed, the OB size and cell area are different between high- and low-OC materials [19,23,24]. Apart from that, some unusually large OBs in the seeds of low OC varieties have also been observed in *B. napus*, which were presumed to be related to the low accumulation of oleosins [25]. In a subsequent study, the OB to cell-area ratio of high-oil rapeseed was significantly higher than that of rapeseed with low OC [19]. However, Dong et al. suggested that there were smaller OB sizes and a larger number of OBs in HOC rapeseed [26].

OBs are essential for seed-oil accumulation, and study of the molecular mechanism of OB formation and morphogenesis during seed development is necessary for the improvement of oil content in rapeseeds. OB formation in seeds starts with the synthesis of fatty acids in plastid, which is then transferred to the endoplasmic reticulum (ER) for the synthesis of TAG, and at last, the TAG and OB proteins together constitute the OBs [27,28]. OBs can be detected at the heart-shaped embryo stage, after which they begin to aggregate or divide, and eventually form into a stable structure [29]. By comparing protein expression differences between two rapeseed cultivars with different OC, 32 OB proteins were identified [30]. These differentially expressed proteins were found to coincide with the appearance of OB in the seeds, and their function is conserved among diverse species [30,31]. There are many types of OB proteins, and the number of OBs is also varied in different species. For example, there are 19 OB proteins in *Saccharomyces cerevisiae* [32], eight oleosins in *Carthamus tinctorius* [33], 46 OB proteins in Arabidopsis [34], and 48 oleosins in *B. napus* [35]. Analogs with similar OB-protein structures also have been found in *Pinus massoniana*, *Nannochloropsi* ssp., *Jatropha curcas*, and *Paeonia ostii* [18,36,37,38,39,40].

OB-related proteins play a regulatory role in OB genesis, morphological construction and OB transport. Oleosins can maintain the integrity of OB and improve cold resistance by preventing OB adhesion [5,11,22,41,42]. All five oleosin proteins (OLE1-OLE5) are involved in protein modifications (such as phosphorylation and ubiquitination before oil degradation), and it has been suggested that toleosin proteins have an important role in regulating the dynamics of oil and lipids [43]. It was also found that OLE1 could positively enhance oil accumulation and OB size by affecting the TAG metabolism [44]. OBAP1 is an oleosome-associated protein that is not located on the surface of the OB in maize, but is an oleosome-related protein present in the cytoplasm, which is involved in the regulation of the synthesis of OB proteins on the ER to the formation of the OB [45,46]. Oleosins and caleosins are accumulated during early seed developmental stages (12–17 days after pollination), while the steroleosins are accumulated in later stages (~25 days after pollination) in *B. napus* [46,47]. OsHSD1 is a steroid dehydrogenase that is localized in OB, it was found that mutation of this gene could increase the content of very long-chain fatty acids (VLCFAs) [48]. Although some clues about the process and dynamics of OB development have been found through the isolation and analysis of OB-related proteins [23,25,29], the early developmental factors and control processes of OB morphogenesis are still unclear [19,25,29]. Apart from that OB proteins might regulate OB formation, other genes involved in lipid metabolism also affect OB size [42]. For example, LEC1 and LEC2 are two important regulators in seed maturation and oil accumulation, which could promote the expression of five specific genes encoding oleosins [49,50].

Starch is a storage carbohydrate widely synthesized in plants and is composed of homopolymers of glucose [51,52,53]. Fructose-6-phosphate (Fru6P) is an intermediate of the Calvin Cycle, which was converted to ADP-Glucose (ADPGlc) by phosphoglucose isomerase (PGI), plastidial phosphoglucomutase (PGM1) and ADP-glucose pyrophosphorylase (AGPase) [54,55,56]. A portion of ADPGlc is produced as a branched chain starch by soluble starch synthase (SSS), starch-branching enzyme (SBE) and starch isomerase (ISA), while the other portion is produced as a straight chain starch by granule-bound starch synthase (GBSS) [57,58,59]. Starch is stored in cells in the form of starch granules (SG). SGs are present in all parenchyma cells, especially in various storage organs, such as the endosperm and cotyledon of seeds [51].

At present, the majority of studies on the relationship between OB morphology and seed OC have focused on mature seeds, and the changes of OB in whole seed development stages are still unclear. The morphogenesis and development of OBs in the early stages of embryos may have a fundamental influence on the quantity and morphology of the final OB formation. To shed light on the OB dynamics in seed development, transmission electron microscope (TEM) analysis was performed on the developing seeds in *B. napus* with HOC (N53-2, the OC was about 50%) and LOC (Ken-C8, the OC was about 39%). Combined with the transcriptomic analysis of the developed seeds, most of the genes involved in malonyl-CoA metabolism, fatty acid carbon chain extension, lipid synthesis, and starch synthesis were more highly expressed in the materials with HOC than in that of the materials with LOC. These results will help us to better understand the dynamic changes of OB during seed development in *B. napus*, and will also can provide new clues for improving the OC in rapeseed in the future.

## 2. Results

### 2.1. Differences of OBs in HOC and LOC Rapeseed during Seed Development

**Figure 1:**
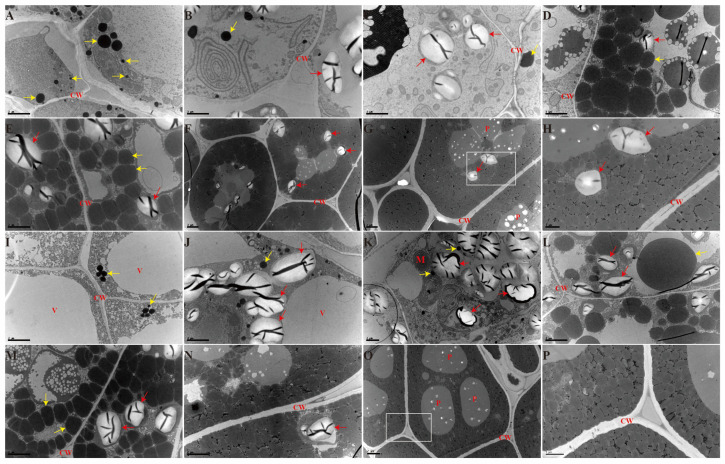
Dynamics of oil bodies in different seed development stages in HOC and LOC materials. (**A**–**G**) TEM images of seeds taken at 10, 17, 24, 30, 36, 42 and 48 days after fertilization in N53-2, respectively; bar = 2 µm; (**H**), an enlargement of the area indicated by the white box in (**G**). bar = 1 µm; (**I**–**O**), TEM images of seeds taken at 10, 17, 24, 30, 36, 42 and 48 days after fertilization in Ken-C8, respectively; bar = 2 µm; and (**P**), an enlargement of the area indicated by the white box in (**O**). bar = 1 µm. H1, H2, H3, H4, H5, H6, and H7 indicated seeds taken at 10, 17, 24, 30, 36, 42 and 48 days after fertilization in N53-2, respectively. L1, L2, L3, L4, L5, L6, and L7 indicated seeds taken at 10, 17, 24, 30, 36, 42 and 48 days after fertilization in Ken-C8, respectively. The red arrows show starch granules, and the yellow arrows show oil bodies. M, mitochondrion; V, vacuole; P, protein body; CW, cell wall.

**Figure 2:**
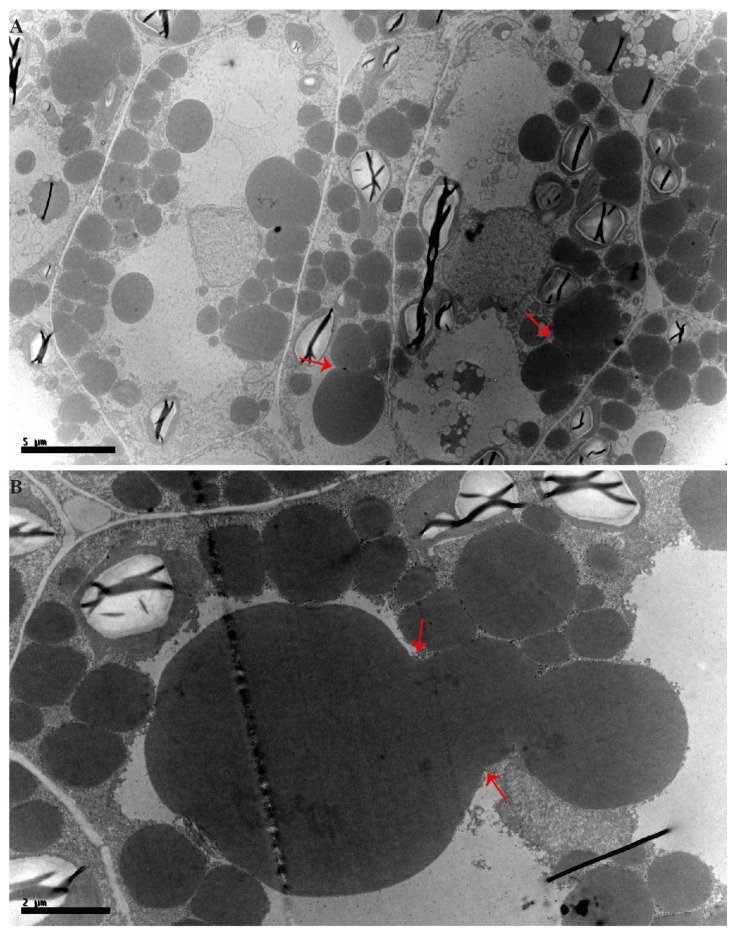
Oversized oil bodies within developing seeds of HOC and LOC materials: (**A**) TEM images of seeds taken 30 days after fertilization in HOC materials, bar = 5 µm; and (**B**) TEM images of seeds taken 30 days after fertilization in LOC materials, bar = 2 µm. Red arrows indicate that oil body aggregation might occur in developing seeds.

**Figure 3:**
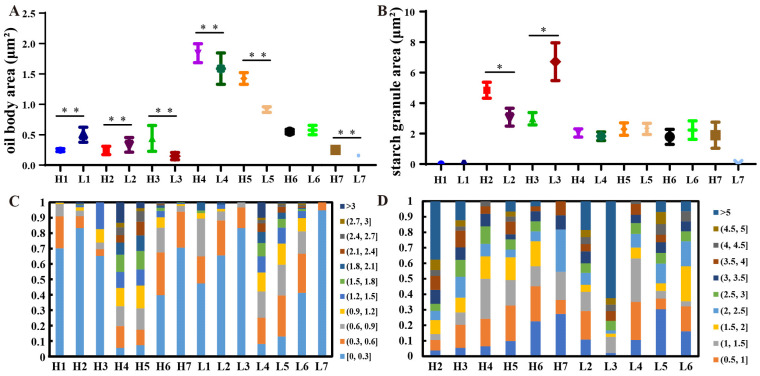
Differences in oil body and starch granule size in seeds at different developmental stages of HOC and LOC materials: (**A**), boxplot of oil body size for seeds at different stages. **, *p* < 0.01; (**B**) boxplot of starch granule size for seeds at different stages. *, *p* < 0.05; and (**C**), distribution of different sizes of oil bodies in developing seeds of N53-2 and Ken-C8. (**D**), distribution of different sizes of starch granules in developing seeds of N53-2 and Ken-C8. H1, H2, H3, H4, H5, H6, and H7 indicated seeds taken at 10, 17, 24, 30, 36, 42 and 48 days after fertilization in N53-2, respectively. L1, L2, L3, L4, L5, L6, and L7 indicated seeds taken at 10, 17, 24, 30, 36, 42 and 48 days after fertilization in Ken-C8, respectively.

First, the seeds of N53-2 (HOC) and Ken-C8 (LOC) in different developmental stages were collected for OC analysis. It was found that the OC grew slowly at the early stages, then rapidly increased in the middle stages, and finally reached a plateau (Figure S1). The TEM analysis revealed that the average size of OBs increased at first and then decreased with the seed development in both materials. The average OB size was the largest in stage 4, with the 1.84 ± 0.08 and 1.58 ± 0.13 μm^2^ in N53-2 and Ken-C8, respectively. Apart from that, the larger OBs were also observed in stage 4, with 14.39 μm^2^ and 52.32 μm^2^ in H4 and L4, respectively (Figure 1, Table 1). In other stages, the largest OB size was less than 8 μm^2^. Further analysis showed that these large OBs might be formed by the aggregation of multiple OBs (Figure 2). Considering the different distributions of OB size, the average OB size was mainly affected by relatively smaller OBs. The proportion of OBs smaller than 0.3 μm^2^ first increased and then decreasedm in contrast to the trend of the average OB size, and the lowest proportion was found in stage 4 (5.6% and 8.1% was in H4 and L4, respectively) (Figure 3A,C).

The differences of OB size between N53-2 and Ken-C8 at different developmental stages were also analyzed. At stages 3, 4, 5, and 7, the average OB size was significantly higher (*p* < 0.05) in N53-2 than that of Ken-C8 (Figure 3A). However, this was reversed at stages 1, 2, and 6 (Figure 3A). Especially in stage 1, the average OB size of N53-2 was about half of tKen-C8, but the number of OBs in H1 was much higher than that of L1. OB size also varied greatly in that of L1 (Figure 1A, I). The average OB size in both materials at stage 7 was lower than that of stage 6, and N53-2 was 62% higher than that of Ken-C8. Consistent with these results, the percentage of OBs with sizes less than 0.3 μm^2^ in N53-2 was less than that of Ken-C8 in stages 3, 4, 5, and 7. For example, 70.2% of the OBs is less than 0.3 μm^2^ in H1, while only 47.4% of the OBs was less than 0.3 μm^2^ in L1. However, in H7, 70.6% of the OB area was less than 0.3 μm^2^, while 94.8% of the OB area in L7 was less than 0.3 μm^2^. In summary, the distribution of OB dimensions differed at different stages of seed development between the materials with HOC and LOC.

### 2.2. Difference of SGs in HOC and LOC Rapeseed during Seed Development

In addition to OBs, starch granules (SGs) are another important structure in the developing seeds, and exist as semi-crystalline granules in seeds. In the present study, SGs were detected in all stages except for stage 1 (Figure 1). The number of SGs in stage 2 was the largest in both HOC and LOC rapeseeds. It was revealed that the number of SGs gradually decreased with the seed development, and which could also be seen in some cells of the mature seeds. In HOC rapeseed, the largest SG was detected in H2, with an area of 4.84 μm^2^, while the largest SG area was detected in L3, with an area of 6.72 μm^2^ in Ken-C8 (Figure 3B, Table 1). In stage 2, the average area of SGs in HOC rapeseed was significantly higher than that of the LOC rapeseed, while the average SG area of HOC rapeseed was significantly lower than that of LOC rapeseed in stage 3 (Figure 3B). The average area of SGs in H2, H3, L2 and L3 was more than 3 μm^2^. At other stages, the average area of the SGs was about 2 μm^2^ and no significant difference was found in these stages between HOC and LOC rapeseed (Figure 3D). Further analysis showed that the percentage of SGs with an area greater than 5 μm^2^ was 37.59% and 18.46% in H2 and L2, 12.16% and 62.5% in H3 and L3, respectively, but were rare in the other stages (Figure 3D). In summary, the difference of the oversized SGs resulted in the difference between HOC and LOC.

### 2.3. The Difference of Cell Space and OB Area to Cell Area Ratio in HOC and LOC Rapeseed

**Figure 4:**
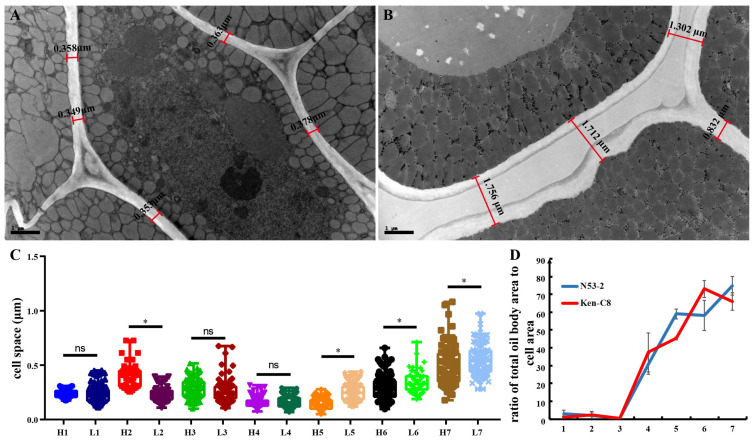
Difference of cell space and oil body area to cell area ratio in HOC and LOC materials: (**A**) TEM images of seeds taken 48 days after fertilization in N53-2, bar = 1 µm; (**B**) TEM images of seeds taken 48 days after fertilization in Ken-C8, bar = 1 µm; (**C**) boxplot of cell space for seeds at different stages; *, *p* < 0.05; ns, no significant difference; and (**D**) variation in the ratio of total oil body area to cell area within developing seeds of N53-2 and Ken-C8.

The proportion of the total area of OBs in the cell was one of the main influencing factors for the OC. It was revealed that the proportion of OBs within the cells was extremely low in the first three stages, after which they increased significantly (Figure 4D). Further analysis showed that the proportion of OBs was different in HOC and LOC materials. In stages 5 and 7, the proportion of OBs in HOC rapeseed was higher, occupying ratios of 59.03% and 74.81%. Otherwise, the ratio in LOC rapeseed was 45.28% and 66.04%, respectively. In stages 4 and 6, the proportion of OBs was higher in LOC rapeseed, which occupied ratios of 37.44% and 73.01%; otherwise, the ratio in HOC rapeseed was 30.85% and 50.06%, respectively. In HOC rapeseed, the proportion of OBs was the highest in H7, while the proportion of OBs in LOC rapeseed reached the highest value in L6, which might be due to the β oxidation of fatty acids that occurred after this stage in LOC rapeseed.

The cell space of developing seeds in the *B. napus* of HOC and LOC was also measured. It was revealed that no significant difference between N53-2 and Ken-C8 was found in the early stages of seed development. Otherwise, in the later stages of seed development, a significant difference in the cell space in HOC rapeseed and LOC rapeseed was observed (Figure 4A,B). At stage 7, the average cell space of H7 was 0.48 ± 0.18 μm, which was 14% lower than that of L7 (0.55 ± 0.14 μm) (Figure 4C).

### 2.4. Differential Expression of Genes Involved in the Lipid Regulation and Starch Accumulation

**Figure 5:**
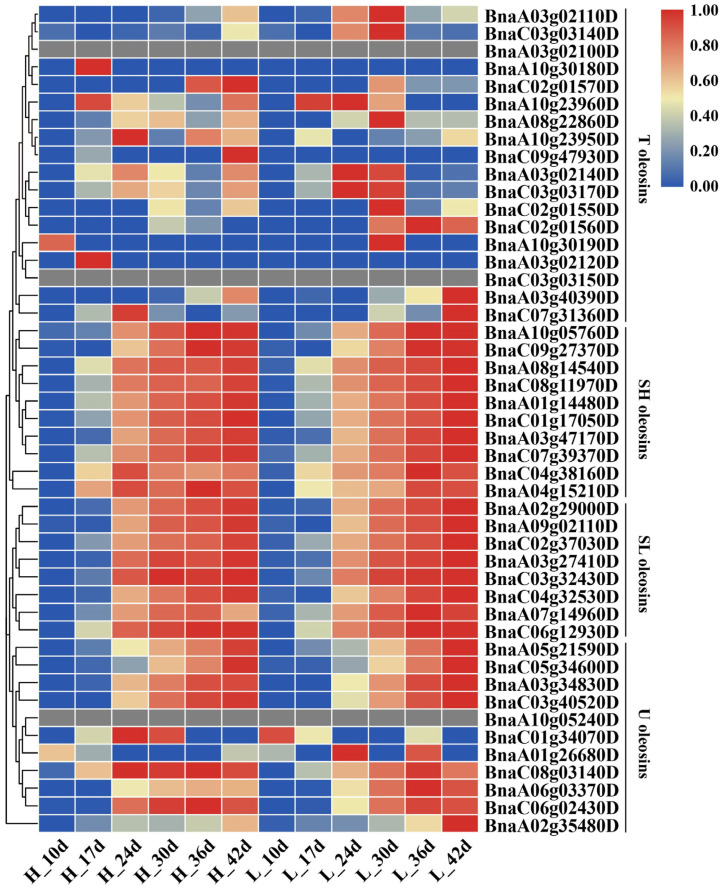
Heatmap of the oleosin gene family in HOC and LOC materials. The colors correspond to the value of FPKM, ranging from blue (low expression) to red (high expression).

The gene expressions related to oil synthesis (e.g., *GPAT4*, *LPAT2*, *DGAT*), OB assembly (e.g., oleosin, caleosin, steroleosin) and starch metabolism (e.g., *PGI, PGM, APL*) were analyzed. The expression analysis of the three main proteins (oleosin, caleosin and steroleosin) showed that all the oleosin proteins were highly expressed in the middle and late stages of seed development, except for T oleosins (Figure 5). Further analysis revealed that the expression of all SH and SL oleosins increased gradually during seed development. Apart from oleosins, other OB-associated proteins, such as caleosin, steroleosin were also analyzed. It was revealed that the expression trends of some steroleosins and OB-associated proteins were similar to that of SH oleosins and SL oleosins (Figure S2). However, the expression trend of PAP- and LD-associated proteins was opposite to that of other proteins, which were highly expressed in the early stage of seed development and decreased in the late stage of seed development (Figure S2).

**Figure 6:**
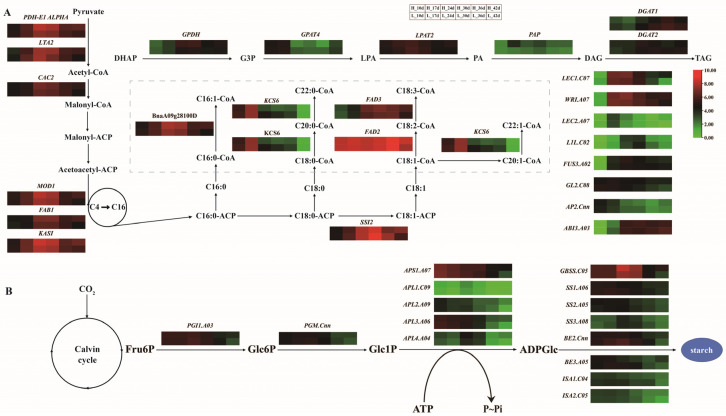
Expression of key genes in pathways of lipid metabolism (**A**); and starch metabolism (**B**). The colors correspond to the value of FPKM, ranging from green (low expression) to red (high ex-pression).

Lipid synthesis and accumulation are regulated by multiple transcription factors (TFs), such as LEC1, LEC2, L1L, WRI1, GL2, AP2, ABI3 and FUS3. It was shown that LEC1, WRI, FUS3, GL2 and ABI3 were highly expressed during seed development, while LEC2, L1L and AP2 were expressed with high specificity during the period. Except for L1L and ABI3, almost all TFs were expressed more highly in HOC rapeseed than in that of LOC rapeseed (Figure 6). At 30 DAF, the expression of L1L in LOC rapeseed was 58% higher than that of HOC rapeseed, and the expression of ABI3 in LOC rapeseed was 45.6% higher than that of HOC rapeseed at 42 DAF. The results indicate that these TFs might have different regulatory mechanisms on lipid formation at different developing periods.

In addition to TFs, the expression of key genes involved in lipid synthesis and transport was also analyzed (Figure 6A). It was shown that the genes related to malonyl CoA metabolism and fatty acid carbon chain extension were highly expressed during whole seed development, especially in the middle development stages. In KCS6, key genes for very long-chain fatty acid synthesis were highly expressed in early seed development. Fatty acid dehydrogenase, such as FAD3 and SIS2 was highly expressed in middle and later seed development, whereas FDA2 was highly expressed throughout seed development. Compared with the above genes, the expression of GPAT, LPAT2 and DGAT1, key genes of the Kennedy pathway, was relatively lower. LPAT showed high expression throughout seed development, while GPAT was highly expressed in the early stages and DGAT was expressed in the late stages. The expression of most key genes for lipid synthesis was higher in HOC rapeseed; however, the expression trend of DGAT was reversed, and it was higher in LOC rapeseed.

Genes that regulate starch synthesis were also analyzed (Figure 6B). It was revealed that both PGI and PGM were highly expressed at the early stage of seed development, and then expression levels decreased gradually. Some AGPase genes were highly expressed in the early stage of seed development, while others (such as APL4), were highly expressed in the middle stage of seed development. Genes that directly control starch synthesis are redundant in rapeseed, and their expression patterns were also different. For example, GBSS had a very high expression level in early development, while some genes such as ISA1 and ISA2 were expressed at low levels throughout the process. Except for BE2, the expression of starch synthesis genes was higher in HOC rapeseed, but the expression of BE2 in LOC rapeseed was almost twice that in HOC rapeseed at 24 DAF. Quantitative RT-PCR results indicated that expression trends of these genes in extremely high and low OC rapeseed were consistent with those in developing seeds of HOC and LOC rapeseed (Figure S3).

## 3. Discussion

Increasing the oil content is one of the main goals of rapeseed breeders, and the OB is of interest to scientists because of its relationship to oil content [6,20]. OBs are organelles that store TAG in the cytoplasm and play a vital role in seed development and germination [6,20]. Prior studies have noted the importance of the relationship between OB and OC, and many studies have investigated the shape and distribution of OBs with different oil contents in *B. napus* [19,23,24,27]. However, the dynamic development of OBs during embryogenesis is still unclear [19,25,29]. A previous study that focused on the developmental embryos in *B. napus* found that the OBs could be observed in the heart-shaped embryo (15 days after pollination) [23,29]. In the present research, OBs could be observed in H1 and L1, indicating that the presence of OBs appeared before the heart-shaped embryos, and that TAG was also stored in OBs in early embryos. In tung trees, it was found that the size of OBs in endosperm gradually decreased with the increase of OC, but the number of OBs was increased [60]. In diatoms, the size and number of OBs were varied in species during lipid accumulation [61]. The present results showed that the average size of OBs increased at first and then decreased with seed development in both HOC and LOC *B. napus* materials, and this indicated the difference in OB aggregation during seed development. In early embryos, the number of OBs in cells is small, most of the OBs are round, and the distance between any OB is relatively long. With the increase of OC, the number of OBs and the total area of OBs also began to increase, and the distance between OBs began to decrease, which might lead to the occurrence of irregularly shaped OBs and OB aggregation.

The size and the distribution of OBs might reflect the OC, and the OB size in the materials with different OC has always been of concern in *B. napus* [19,24,25]. Generally, the diameter of mature OB ranges from 0.5 µm to 2.5 µm [28]; otherwise, a wider range of OB sizes was observed in developing seeds of N53-2 and Ken-C8, which indicated the dramatic changes of OB size in seed development. A strong relationship between unusually large OBs and LOC has been reported in rapeseed [25]. In the present research, although the largest OB (52.322 μm^2^) was found in the L4 stage, the ratio of relatively larger OB in HOC was more than that in LOC in almost all periods. On the other hand, it was also found that the average OB size of N53-2 (HOC) and Ken-C8 (LOC) was 0.25 and 0.15 μm^2^ in mature seeds, respectively. This is in accordance with earlier observations in maize [16], which showed that *B. napus* with HOC might possess relatively larger OBs than that of *B. napus* with LOC, but this trend was not consistent during seed development. Previous studies agreed that the OB to cell-area ratio was higher in HOC materials [23,24,26]. Similar results were also found in the present research, where the total OB area of the HOC material accounted for 74.8% of the cell area, which was higher than that of the LOC material. The 3D structure of LDs was also reconstructed, and it was found that the cell space in HOC rapeseed was much shorter than that of LOC rapeseed [62]. An extremely large cell space was also detected in LOC material in this study. Previous research also verified this conclusion [24], which indicates that the larger cell space leads to lower cell density, and then results in the lower oil content of *B. napus*. These findings provide some new ideas on how to improve oil content in future.

The hemi-unit membranes of OBs were composed of monolayers of polar lipids or phospholipids (PL) surrounded by oleosin, caleosin and steroleosin. Oleosin and caleosin accumulate around the torpedo-shaped embryo period [46,47]. These structural proteins not only maintain the stability of the OB structure but also affect TAG metabolism and OB size [44]. In the present study, the expression of these genes was analyzed, and it was found that three classes of oleosin proteins (except for T oleosin) were mainly expressed in seeds. The gene expression of all SH oleosins and SL oleosins was increased gradually during seed development. With the increase of lipid accumulation, the number of OBs was gradually increased, which require more structural proteins to maintain the stability of OBs. Therefore, most OB proteins are highly expressed in late seed development. It was also found that a lack of oleosins could cause the OB to compress and fuse, which resulted in an enlarged OB in the middle stage of seed development in Arabidopsis [6,7,41]. Increased oleosin might result in OB aggregation, conforming the phenotype of the average OB size decrease from stage 4 to stage 7. Apart from OB structure proteins, some genes involved in lipid synthesis and transcriptional regulation could also affect OB size [49,63,64], and the important functional enzymes and TFs showed higher expression levels in N53-2 with HOC than those of Ken-C8 with LOC, except for L1L, ABI3 and DGAT.

Starch is a storage carbohydrate widely synthesized in plants and SGs are the main storage form of starch in developing seeds [51,53]. Previous research showed that Cr stress could increase the size of starch grains in the leaf mesophyll cells of rapeseed [65], but few studies have reported on the question of SG dynamics in the seed development of rapeseed. In the present study, the SG could be detected in most periods of the developing seeds, and the number of SGs gradually decreased with seed development. In higher plants, Fructose-6-phosphate (Fru6P) was converted to starch by PGI, PGM1, AGPase, and starch synthase [54,55,56]. It was shown that most of these vital genes were highly expressed in early seed development, and then decreased gradually with the development of seeds, which was consistent with the variation of SG numbers. Compared with these genes, genes associated with carbon-chain extension were more highly expressed in the middle and late stages. The peak size of SGs was earlier than that of OBs. These results might indicate that large amounts of starch were synthesized in early seeds to provide energy for fatty acids synthesis in the later stages of seed development.

In conclusion, the changes of OB and SG size were described between HOC and LOC during seed development. Combined with the transcriptome of the developed seeds, the primary mechanism was revealed for OB formation. This study will help us to better understand the dynamic changes of OB during seed development in rapeseed, and provide new clues for improving the OC in rapeseed in future.

## 4. Materials and Methods

### 4.1. Plant Material and Growing Conditions

Two rapeseed materials, N53-2 and Ken-C8, with relative HOC of about 50% and relative LOC of 39% were used. The experimental materials were planted at Huazhong Agricultural University. Planting was performed with four rows per material and 12 plants per row, with plant spacings of 10 cm and row spacings of 30 cm [66]. The field management was carried out according to local conventional production methods. After fertilization, two materials with HOL and LOC were marked simultaneously, and then the siliques were harvested at 10, 17, 24, 30, 36, 42 and 48 days after fertilization, and named as H1, H2, H3, H4, H5, H6, H7 and L1, L2, L3, L4, L5, L6 and L7, respectively. Some siliques were ground in liquid nitrogen and then stored in a refrigerator at −80 degrees and used for transcriptome analysis and qRT-PCR. The ovules of the other part of the siliques were separated and then fixed using a 2.5% glutaraldehyde solution, then used for TEM analysis.

### 4.2. Measurement of Oil Content

The method for oil content measurement was according to the method previously reported with some modifications [67]. Seeds were removed from siliques manually before freeze-drying and thirty milligrams of dry seeds were used for oil content analysis. After placing the seeds into a glass tube, 2 mL of 2.5% sulfuric acid-methanol solution, 0.8 mL of toluene, and 0.4 mL of a 2 mg/mL C17:0 solution in toluene were added. The mixture was vortexed and then heated in a 90 °C water bath for 1 h, and then 3.6 mL ddH_2_O and 2 mL hexane were added after the tube had cooled. The supernatant was filtered using a 0.45 μm microporous membrane after standing overnight. The filtrate was used to determine the fatty acid content by using an Agilent 7890A instrument. At least three replicate samples were examined for all the experiments.

### 4.3. Measurement of Oil Body Sizes, Starch Granule Size and Cell Space

The method for TEM analysis was conducted according to the method previously reported, with minor modifications [24]. The ovules were manually isolated and fixed with 2.5% glutaraldehyde in a 0.1 M phosphate buffer (pH 6.8), and then fixed with 1% OsO4. These samples were then dehydrated with gradient acetone (20%, 50%, 70%, 90% and 100% × 3) and embedded with epoxy resin. The samples were then cut into 70 nm slices using a slicer (Leica EM UC7, Wetzlar, Germany), collected into a copper mesh and then stained with supersaturated uranyl acetate and 0.4% lead citrate. Samples were then observed and imaged using a transmission electron microscope (HT7700, Tokyo, Japan).

ImageJ was used to open the TEM image and then set the scale of the image [68]. The freehand selection tool was used to trace the shape of OBs, SGs and cells, and then the measuring tool was used to analyze the area of the OBs, SGs and cells. The area of all the OBs within a cell can be summed to obtain the total OB area and thus calculate the total OB area as a proportion of the cell area. For cell space, the straight tool was used to draw straight lines between two cells that were evenly spaced, and then the measuring tool was used to analyze the length. At least 10 TEM images were used for analysis at every stage. Statistical differences between different samples were evaluated using *t*-test, with results *p* < 0.05 indicating significant differences between the two samples.

### 4.4. Gene Expression Analysis

The developing seeds were harvested and the RNA-seq analysis was performed at 6 periods from extremely HOC and LOC materials from the KN DH population derived from the hybridization between N53-2 and Ken-C8 [69]. Based on these data, we explored the expression levels of some known important genes involved in malonyl-CoA metabolism (e.g., *LTA2*, *CAC2*), fatty acid carbon chain extension (e.g., *MOD1*, *FAB1*, *KAS1*, *KCS6*) and lipid synthesis (e.g., *GPAT4*, *LPAT2*, *DGAT*). In addition to this, the expression of OB-associated proteins and some important transcription factors regulating lipid metabolism were also analyzed.

### 4.5. RNA Extraction and Quantitative RT-PCR

The total RNA of developing seeds was extracted using the RNAprep Pure Plant Plus Kit (Tiangen Biotech Co., Ltd. Beijing, China) according to the instruction manual. First Stand cDNA was synthesized using HiScript^®^ III 1st Strand cDNA Synthesis Kit (Vazyme Biotech Co., Ltd. Nanjing, China). Quantitative RT-PCR was performed with Hieff^®^ qPCR SYBR Green Master Mix (Cat No.11203ES08; Yeasen, Shanghai, China). The relative expression of target genes at different developmental stages was measured according to the method of 2^−ΔΔCt^ and ACTIN was used as the internal control. All the samples were tested in three biological replicates.

## Figures and Tables

**Table 1:** Statistical value of oil body and starch granule size for HOC and LOC materials in 7 periods.

	Oil Body					Starch Granule				
	Minimum (μm^2^)	Median (μm^2^)	Maximum (μm^2^)	Mean (μm^2^)	Std. Error of Mean(μm^2^)	Minimum (μm^2^)	Median (μm^2^)	Maximum (μm^2^)	Mean (μm^2^)	Std. Error of Mean (μm^2^)
H1	0.005	0.181	1.258	0.246	0.01217	ns	ns	ns	ns	ns
H2	0.013	0.0995	5.663	0.2408	0.03448	0.384	4.17	12.93	4.843	0.2639
H3	0.018	0.259	1.409	0.4393	0.1025	0.296	2.647	7.208	2.974	0.2032
H4	0.087	1.325	14.4	1.841	0.07905	0.495	1.758	4.343	2.039	0.1322
H5	0.068	1.301	5.412	1.426	0.04908	0.517	1.798	6.631	2.293	0.2041
H6	0.006	0.396	3.95	0.5491	0.01979	0.349	1.587	6.548	1.784	0.2422
H7	0.012	0.183	1.67	0.2489	0.008478	0.371	1.4	4.194	1.897	0.3845
L1	0.022	0.307	2.052	0.4998	0.06168	ns	ns	ns	ns	ns
L2	0.014	0.189	6.643	0.3333	0.0609	0.382	2.418	10.19	3.075	0.2913
L3	0.014	0.069	0.624	0.1473	0.02935	0.613	5.739	15.87	6.716	0.6154
L4	0.007	1.092	52.32	1.587	0.1314	0.485	1.4	4.315	1.823	0.141
L5	0.035	0.7335	3.759	0.9128	0.02376	0.072	2.23	7.293	2.309	0.1858
L6	0.033	0.3895	7.737	0.5756	0.03918	0.238	2.041	7.283	2.228	0.2994
L7	0.024	0.149	0.431	0.153	0.004933	ns	ns	ns	ns	ns

ns indicates unmeasured valid data.

## Data Availability

All data was enclosed in the main text and Supplementary Information Files. Any detailed datasets generated during and/or analyzed during the current study are available from the corresponding author on reasonable request.

## Supplementary Materials

The following supporting information can be downloaded at: https://www.mdpi.com/article/10.3390/ijms24044201/s1.
