# Supplementary material for: Characterization of Oil Body and Starch Granule Dynamics in Developing Seeds of Brassica napus
Source: Int J Mol Sci. 2023 Feb 20;24(4):4201. doi: 10.3390/ijms24044201 (PMC9967339; doi:10.3390/ijms24044201)
Supplement: Supplementary file 1 [file ijms-24-04201-s001.zip › ijms-2192209-supplementary.pdf]

Characterization of Oil Body and Starch Granule Dynamics in Developing Seeds of *Brassica napus*

Kang Chen, Yongtai Yin, Yiran Ding, Hongbo Chao and Maoteng Li

Supplementary Material

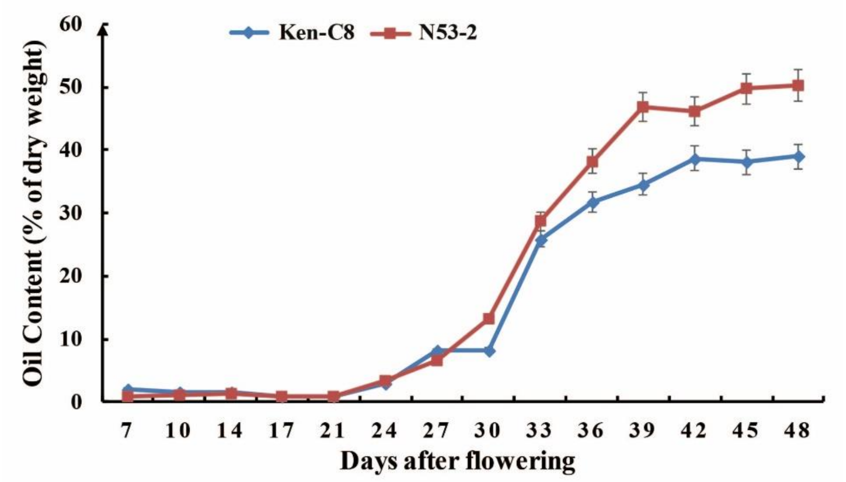

Figure S1: The oil content of developing seeds from HOC material N53-2 (50%) and LOC material Ken-C8 (39%).

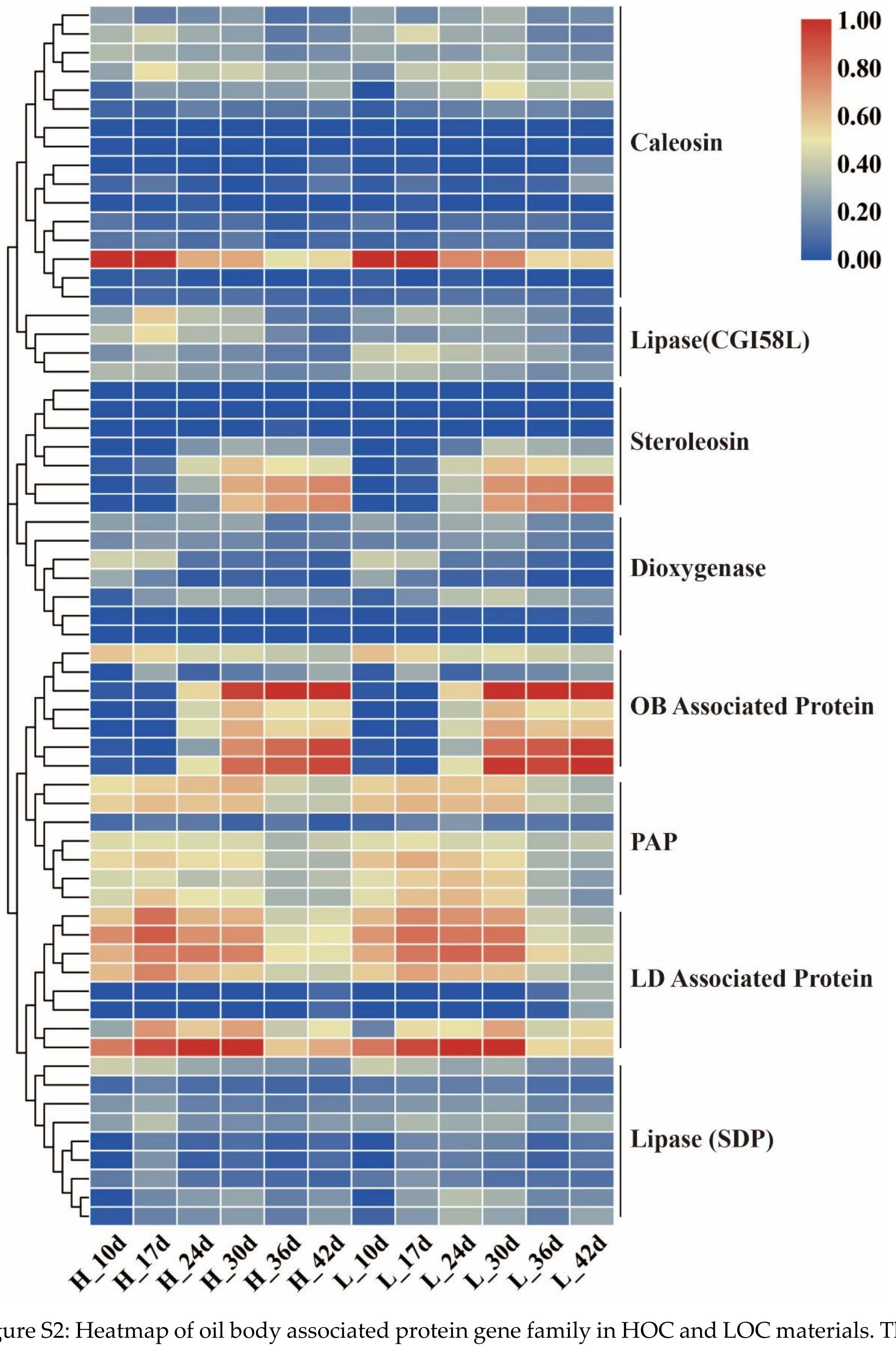

Figure S2: Heatmap of oil body associated protein gene family in HOC and LOC materials. The colors correspond to the value of FPKM, ranging from blue (low expression) to red (high expression).

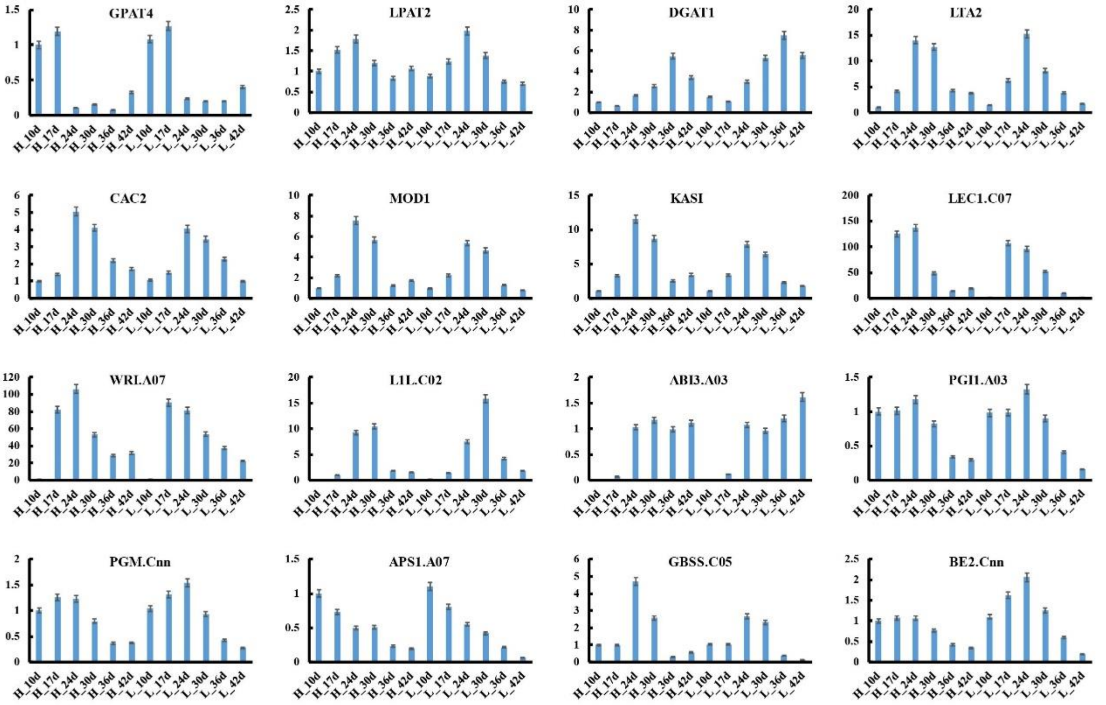

Figure S3: Expression of genes involved in lipid and starch metabolism in developing seeds from HOC and LOC materials. H_10d, H_17d, H_24d, H_30d, H_36d, and H_42d indicated seeds were taken at 10, 17, 24, 30, 36, and 42 days after fertilization in N53-2, respectively. L_10d, L_17d, L_24d, L_30d, L_36d, and L_42d indicated seeds were taken at 10, 17, 24, 30, 36, and 42 days after fertilization in Ken-C8, respectively.
